# From Initial to Situational Automation Trust: The Interplay of Personality, Interpersonal Trust, and Trust Calibration in Young Males

**DOI:** 10.3390/bs16020176

**Published:** 2026-01-26

**Authors:** Menghan Tang, Tianjiao Lu, Xuqun You

**Affiliations:** 1 Shaanxi Provincial Key Laboratory of Behavior and Cognitive Neuroscience, School of Psychology, Shaanxi Normal University, No. 199, South Chang’ an Road, Yanta District, Xi’an 710062, China; 2 Student Mental Health Education Center, Northwestern Polytechnical University, Xi’an 710062, China

**Keywords:** trust in automation, situational trust, interpersonal trust, personality traits, automated driving, eye tracking, mental workload, human–machine interaction

## Abstract

To understand human–machine interactions, we adopted a framework that distinguishes between stable individual differences (enduring personality/interpersonal traits), initial trust (pre-interaction expectations), and situational trust (dynamic calibration via gaze and behavior). A driving simulator experiment was conducted with 30 male participants to investigate trust calibration across three levels: manual (Level 0), semi-automated (Level 2, requiring monitoring), and fully automated (Level 4, system handles tasks). We combined eye tracking (pupillometry/fixations) with the Eysenck Personality Questionnaire (EPQ) and Interpersonal Trust Scale (ITS). Results indicated that semi-automation yielded a higher hazard detection sensitivity (*d′* = 0.81) but induced greater physiological costs (pupil diameter, *ηp*^2^ = 0.445) compared to manual driving. A mediation analysis confirmed that neuroticism was associated with initial trust specifically through interpersonal trust. Critically, despite lower initial trust, young male individuals with high interpersonal trust exhibited slower reaction times in the semi-automation model (*B* = 0.60, *p* = 0.035), revealing a “social complacency” effect where social faith paradoxically predicted lower behavioral readiness. Based on these findings, we propose that situational trust is a multi-layer calibration process involving dissociated attentional and behavioral mechanisms, suggesting that such “wary but complacent” drivers require adaptive HMI interventions.

## 1. Introduction

The rapid development of science and technology has continuously increased and deepened the interaction between humans and machines. Similarly, the importance of trust in automation for human–machine collaboration has become increasingly prominent (Lee & Moray, 1992; Parasuraman & Riley, 1997). The rapid advancement of vehicle automation requires people to perform reasonable task allocations in automatic pilot scenarios to adapt the driver’s attention and cognition to the current needs of the automated system. This requires drivers to maintain an appropriate level of trust in automation (Lee & See, 2004). Drivers who over-trust the automated system may fail to take over for the system in case of failures, which may result in severe accidents. On the other hand, drivers who under-trust the automated system may evade or even refuse to use it (Muir, 1994; Parasuraman & Riley, 1997).

Automation is the execution of certain functions previously performed by humans using a machine agent, such as computers. To clarify the specific roles of drivers and systems, the Society of Automotive Engineers (SAE) J3016 standard defines six levels of driving automation. In manual driving (Level 0), the driver performs all dynamic driving tasks. Semi-automated driving (Level 2) involves partial automation, where the system executes steering and acceleration/deceleration, but the driver must continuously monitor the driving environment and remain ready to intervene immediately. In contrast, fully automated driving (Level 4) enables the system to handle all driving tasks within specific operational design domains, transforming the driver into a passive passenger (SAE International, 2021). However, the user understanding of these distinctions often remains uneven (Gangadharaiah et al., 2023). This implies that the completion of driving operations currently and for the foreseeable future requires the cooperation of automated systems and drivers, necessitating a clear understanding of how trust evolves across these distinct automation levels.

Trust in automation constitutes the foundation of effective human–machine collaboration (Schaefer et al., 2016). However, trust is not a monolithic concept. To ensure conceptual clarity and an alignment with the operational measures used in this study, we adopt a framework that distinguishes between stable individual differences, initial trust, and situational trust (Hoff & Bashir, 2015; McKnight et al., 1998).

First, stable individual differences represent the enduring characteristics of the operator that exist independently of any specific system. In this study, we specifically focus on two key dimensions: personality traits (measured by the Eysenck Personality Questionnaire, EPQ; Eysenck & Eysenck, 1975) and interpersonal trust (measured by the Interpersonal Trust Scale, ITS; Rotter, 1967). We posit that these stable traits serve as the antecedents of trust calibration. Specifically, theoretical models suggest that in the absence of experiential knowledge, individuals with a high general propensity to trust others (high ITS) may project this “faith in humanity” onto machine agents, treating the system as a reliable social actor (Lee & See, 2004; Reeves & Nass, 1996). However, it is also possible that those with high interpersonal reliance may view automation as a distinctly non-human agent, creating a contrast effect.

Second, initial trust refers to the specific attitude an individual forms toward a particular system before active interaction begins. Unlike stable traits, initial trust is a state-based expectation directed at a specific target. In this study, we operationalize initial trust using the Trust in Automation (TIA) scale (Jian et al., 2000) administered prior to the driving task. This measure captures the participants’ baseline expectations of the system’s reliability, which are theoretically projected from their stable individual differences.

Third, situational trust is the dynamic state that evolves during direct interaction with the system, fluctuating based on specific environmental contexts and system performance. This perspective aligns with prior research demonstrating that driver behaviors, such as reaction time, exhibit significant heterogeneity and dependency on the surrounding dynamic context (e.g., in car-following situations) (Tawfeek, 2024). Unlike static questionnaire measures, situational trust manifests in real-time behavioral adjustments. Therefore, this study operationalizes situational trust as a multi-layer calibration process observable through three distinct channels: (1) visual monitoring (fixation duration), based on the premise that higher trust typically reduces the need for active monitoring (Hergeth et al., 2016); (2) physiological cost (pupil diameter), reflecting the cognitive workload and arousal associated with maintaining trust (De Cet et al., 2024); and (3) Behavioral Reliance (reaction time and hazard detection sensitivity).

Despite the extensive literature, critical gaps remain in understanding the pathway from stable traits to dynamic calibration in semi-automated (Level 2) versus fully automated (Level 4) contexts. Previous studies have often struggled to link personality traits to objective handover performance (Molnar et al., 2018). Furthermore, while recent empirical research calls for incorporating physiological data to understand trust (De Cet et al., 2024), few studies have empirically dissociated the “cost” of trust—specifically, whether maintaining situational trust in semi-automation imposes a higher cognitive load. Finally, the specific mechanism by which interpersonal trust relates to automation interaction remains paradoxical; it is unclear whether high interpersonal trust facilitates cooperation or manifests as a dangerous form of “social complacency” (i.e., reducing monitoring effort due to misplaced faith).

Therefore, this study aims to bridge these gaps by conducting a driving simulator experiment comparing manual (L0), semi-automated (L2), and fully automated (L4) conditions. We specifically investigate (1) how stable individual differences (specifically ITS) relate to initial trust expectations and (2) how these factors subsequently are linked to the situational trust calibration process. We hypothesize that semi-automation will induce a “high-effort” monitoring state compared to full automation and that high interpersonal trust may paradoxically predict slower reaction times, indicating a social complacency effect.

## 2. Materials and Methods

### 2.1. Participants

The study participants consisted of 30 males recruited from a local university, with the following inclusion criteria: possess a valid driver’s license and have no prior experience with automated driving or driver assistance systems. The participants’ mean age was 19.8 years (SD = 2.0), ranging from 18 to 26 years, and they had held a driving license for an average of 1.5 years (SD = 0.5). All participants had normal or corrected-to-normal vision. This research was approved by the ethics committee of the local university. All participants were informed of relevant experimental information and provided informed consent before taking part in the experiments.

### 2.2. Material and Apparatus

**Figure 1:**
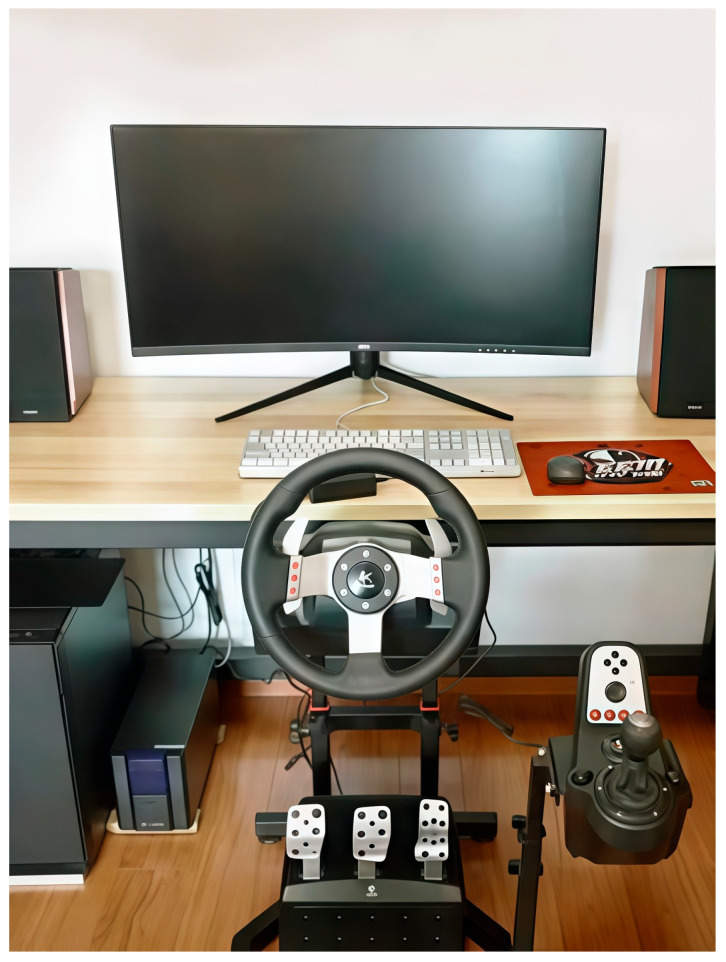
The experimental setup of the driving simulator.

Driving Simulator and Eye Tracking. Experiments were conducted on a desktop computer rendering the driving environment at 60 Hz on an AOC curved ultrawide monitor (2560 × 1080 pixels). The setup included a Logitech G27 driving simulator (Logitech, Lausanne, Switzerland) with a steering wheel, gear shifter, and foot pedals (Figure 1). Eye movements were recorded at 120 Hz using an SMI Eye Tracking System (SensoMotoric Instruments, Teltow, Germany). A 9-point calibration was performed to ensure a mean spatial error of less than 0.8°. Raw data were processed using SMI BeGaze software (Version 3.0, SensoMotoric Instruments, Teltow, Germany), with fixations defined by a dispersion threshold of 100 px and a minimum duration of 80 ms.

Questionnaires. Participants completed three scales. To capture stable individual differences associated with specific trust behaviors, this study used the revised Chinese adult version of the Eysenck Personality Questionnaire (EPQ) (Gong, 1984). This version comprises 88 items, and the subscales demonstrated good reliability in the present study (Cronbach’s *α*: E = 0.86, N = 0.91, P = 0.62, L = 0.76). The Interpersonal Trust Scale (ITS) (Rotter, 1967) was used to assess interpersonal trust as a stable personality trait. We adopted the Chinese translation compiled by Wang et al. (1999), which consists of 25 items (Cronbach’s *α* = 0.70). Finally, the Trust in Automation Scale (TIA) (Jian et al., 2000) was administered to assess participants’ initial trust (i.e., baseline expectations) prior to the system interaction (12 items; Cronbach’s *α* = 0.89).

### 2.3. Procedure

**Figure 2:**
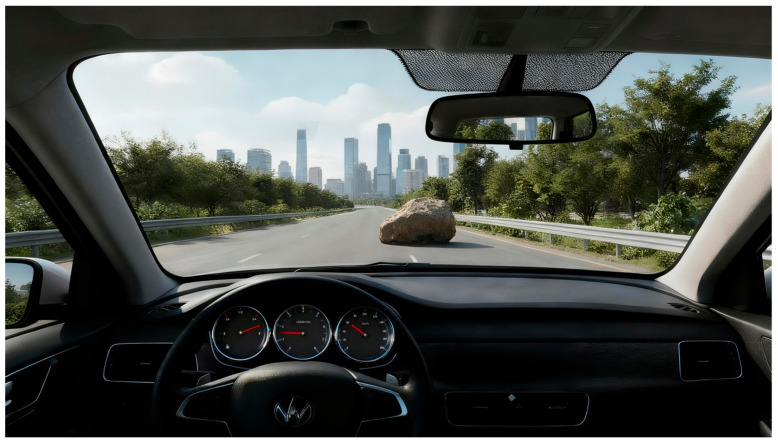
Driving task in manual condition.

**Figure 3:**
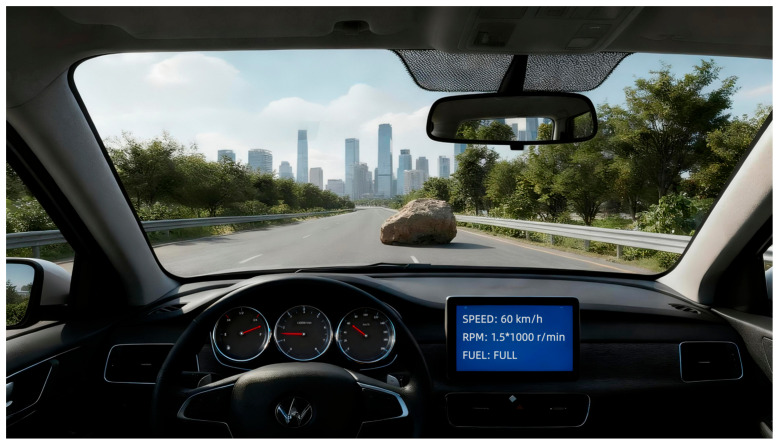
Driving task in semi-automated condition.

**Figure 4:**
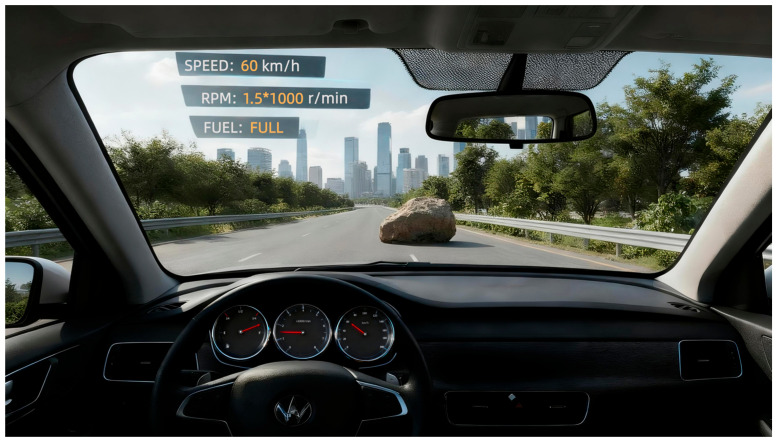
Driving task in fully automated condition.

Experimental Design. The driving task was developed using PsychoPy 3.0. The scenario consisted of a uniform-speed driving task on a highway requiring collision avoidance. Three experimental conditions were designed to align with SAE levels:

- Manual (SAE Level 0): Participants performed all driving tasks manually without system assistance (Figure 2).
- Semi-automated (SAE Level 2): Participants were instructed to keep their hands on the steering wheel and monitor the system via a tablet display located to the right of the mechanical instrument cluster. Scoring penalized false interventions (braking during normal operation) to encourage active monitoring (Figure 3).
- Fully automated (SAE Level 4): Participants were told that the system handled all driving tasks, allowing them to place their hands freely and monitor the system via an AR-based windshield display (Figure 4).

### 2.4. Data Analysis

SPSS 25.0 statistical software was used for data analysis. Conditions are abbreviated as auto (fully automated), manual (manual), and semi (semi-automated) throughout, and braking responses were coded as brake (0/1). The response time (RT) in the behavioral index constitutes the time from the appearance of the obstacle to the participant’s manual braking and was analyzed only in braking trials (brake = 1). To address potential order/practice effects due to the fixed condition sequence, the RT was analyzed using a trial-level linear mixed-effects model (LMM), with condition and centered overall trial order (TrialOverall, centered as trial_c) as fixed effects, participant (ID) random effects, and an AR (1) within-participant residual structure indexed by TrialOverall; Bonferroni-adjusted pairwise comparisons were used for post hoc contrasts.

Signal Detection Theory (SDT) metrics were calculated based on the Hazard Detection Model for the automated conditions only (auto and semi) using all 20 trials per condition. Signal trials (N = 5) were defined as trials with a true hazard (hazard = 1; true automation failure), and noise trials (N = 15) were defined as trials without a hazard (hazard = 0), including trials with system failure cues that were sensor false alarms as well as trials with normal system operation. The SDT contingency table was defined as follows: hit = (hazard = 1 and brake = 1); miss = (hazard = 1 and brake = 0); false alarm = (hazard = 0 and brake = 1); and correct rejection = (hazard = 0 and brake = 0). To handle extreme hit or false-alarm rates (0 or 1), we applied the standard 1/2N correction (Macmillan & Kaplan, 1985), replacing 0 with 1/(2N) and 1 with 1 − 1/(2N) prior to SDT transformations. Because participants were not provided with a numeric failure probability, response bias/criterion indices (β or criterion *c*) were interpreted as a braking/takeover decision criterion (policy) rather than as a direct measure of situational trust. In addition, braking decisions in the automated conditions (auto vs. semi) were analyzed at the trial level using a binomial probit GLMM (“regression SDT”) to test condition differences in sensitivity (d′) while controlling for centered within-block trial order (TrialInCondition, centered as ord_c) and serial dependence.

Raw eye movement data were processed using BeGaze 3.0 (SMI) with a dispersion-based event detection algorithm. For eye tracking, we focused on two measures: overall mean pupil diameter and mechanical-instrument AOI fixation duration (%), defined as the proportion of total fixation duration that fell within the mechanical instrument area of interest (AOI fixation duration/total fixation duration × 100). Condition differences were evaluated using within-subject comparisons with Bonferroni-adjusted pairwise tests.

## 3. Results

### 3.1. Reaction Time (RT) in Braking Trials

Descriptive statistics for braking counts and reaction times are reported in Table 1. Reaction time (RT; seconds) was analyzed at trial level but was restricted to trials in which participants initiated braking (brake = 1), as RT is only defined for braking trials. To address potential order/practice effects arising from the fixed condition sequence (auto → manual → semi), we fitted a linear mixed-effects model (LMM) with automation condition (fully automated, manual, semi-automated) and centered overall trial order (TrialOverall, centered as trial_c) as fixed effects. The model included a random intercept for participant (ID) and a random slope of trial_c by participant. To account for possible serial dependence across repeated trials within participants, TrialOverall was treated as the repeated-measures index within ID, and an AR (1) residual covariance structure was specified.

After controlling for trial order, the effect of condition remained significant: *F*(2, 627.369) = 254.124, *p* < 0.001. Pairwise comparisons showed that manual driving produced faster RTs than fully automated driving (auto–manual = 1.082 s, *SE* = 0.099, 95% CI [0.844, 1.319], *p* < 0.001) and faster RTs than semi-automated driving (semi–manual = 1.054 s, *SE* = 0.100, 95% CI [0.813, 1.294], *p* < 0.001), whereas the auto–semi difference was not significant (auto–semi = 0.028 s, *p* = 1.000; Bonferroni-adjusted). It is worth noting that while the raw means (Table 1) suggested faster RTs in the semi than in the auto condition, this auto–semi contrast was not significant after accounting for overall trial order in the LMM (fixed sequence: auto → manual → semi). Trial order also showed a significant main effect, *F*(1, 236.926) = 6.471, *p* = 0.012, indicating overall practice-related speeding across trials; however, the manual-versus-auto advantage remained robust after accounting for this order trend.

Robustness check. To evaluate the sensitivity to assumptions about within-participant residual dependence, we refit the model using an identity residual covariance structure (assuming independent, homoscedastic residuals within participants). The key inference was unchanged: manual RTs remained faster than fully automated RTs (auto–manual = 1.100 s, *SE* = 0.112, 95% CI [0.832, 1.368], *p* < 0.001), indicating that the manual advantage does not depend on the specific residual covariance specification.

Notably, RT did not differ between the auto and semi conditions, suggesting that the auto–semi contrast is better captured by sensitivity and monitoring indices (Section 3.2 and Section 3.3) rather than braking latency.

### 3.2. Trial-Level Signal Detection Analysis (Auto vs. Semi) Controlling for Order Effects

Descriptive statistics for SDT indices (*d′* and *β*) are reported in Table 2. Because SDT indices were defined for the automated conditions but not for the manual baseline by design, we compared hazard-detection performance between the fully automated and semi-automated conditions using a trial-level “regression SDT” analysis. Specifically, we fitted a binomial GLMM with a probit link (z-space) to braking decisions (brake; 0/1). Both the hazard and condition variables were effect-coded: hazard as −0.5 for noise and +0.5 for signal, and condition as −0.5 for auto and +0.5 for semi. The critical test was the Hazard × Condition interaction. Centered within-block trial order (ord_c; TrialInCondition centered) and its interactions with Hazard × Condition were included to capture potential practice/fatigue trends in both response tendency and sensitivity. The model included a by-participant random intercept, and an AR(1) covariance structure was specified for the repeated measures within participants across the 40 trials.

The hazard (stimulus) effect was significant, *F*(1, 1192) = 40.254, *p* < 0.001, and, critically, the Hazard × Condition interaction was significant, *F* (1, 1192) = 9.483, *p* = 0.002, indicating that sensitivity differed between automation conditions. Interpreting the probit coefficients in SDT terms (at ord_c = 0), sensitivity was higher in the semi condition (*d′* = 0.809) than in the auto condition (*d′* = 0.281), yielding Δ*d′* = 0.528, 95% CI [0.192, 0.865]. In contrast, the main effect of condition was not significant, *F*(1, 1192) = 0.058, *p* = 0.810, indicating no reliable shift in overall response tendency (bias) between auto and semi under this parameterization. Accordingly, the automation-level difference was expressed primarily in sensitivity (Hazard × Condition) rather than in response bias. None of the trial-order terms were significant (ord_c and interactions: *ps* = 0.282–0.997). The estimated AR(1) correlation was small and non-significant (ρ = 0.013, 95% CI [−0.046, 0.072], *p* = 0.664), indicating minimal residual serial dependence; importantly, the key Hazard × condition effect remained significant under this conservative specification.

### 3.3. Eye-Tracking Measures

Because eye-tracking variables were summarized at the condition level (one value per participant per condition), we analyzed them across automation levels (auto, manual, semi) using repeated-measures ANOVAs, with condition as a within-subjects factor. Where the sphericity assumption was violated, Greenhouse–Geisser corrections were applied. Bonferroni-adjusted pairwise comparisons were used for post hoc tests. Descriptive statistics for eye-tracking measures are reported in Table 3.

#### 3.3.1. Overall Pupil Diameter

A repeated-measures ANOVA indicated a significant effect of condition on overall pupil diameter (Greenhouse–Geisser corrected), *F*(1.10, 31.90) = 23.27, *p* < 0.001, *ηp*^2^ = 0.445. The mean (SD) pupil diameter was highest in the auto condition (3.80, 0.50), followed by the semi condition (3.68, 0.51), and lowest in the manual condition (2.84, 1.11). Bonferroni-adjusted pairwise comparisons showed that both auto and semi conditions yielded larger pupil diameters than the manual condition (auto–manual: Δ = 0.96, 95% CI [0.53, 1.40], *p* < 0.001; semi–manual: Δ = 0.84, 95% CI [0.34, 1.34], *p* < 0.001). The difference between the auto and semi conditions was not significant (auto–semi: Δ = 0.12, 95% CI [−0.02, 0.26], *p* = 0.095).

#### 3.3.2. Mechanical-Instrument AOI Fixation Duration (%)

For the mechanical-instrument AOI fixation duration (%), the repeated-measures ANOVA revealed a robust effect of condition (Greenhouse–Geisser-corrected), *F*(1.33, 38.46) = 71.45, *p* < 0.001, *ηp*^2^ = 0.711. The mean mechanical-instrument AOI fixation duration (%) was highest in the semi condition (32.09%, 17.39%), lower in the auto condition (7.26%, 8.10%), and near zero in the manual condition (0.36%, 0.66%). Bonferroni-adjusted pairwise comparisons indicated that all three conditions differed significantly from each other: auto > manual (Δ = 6.90, 95% CI [3.10, 10.71], *p* < 0.001), semi > auto (Δ = 24.83, 95% CI [16.46, 33.20], *p* < 0.001), and semi > manual (Δ = 31.73, 95% CI [23.58, 39.89], *p* < 0.001).

### 3.4. Individual Differences: Trait-Level Predictors and Situational Trust Indicators

#### 3.4.1. Descriptive Statistics and Correlational Overview of Traits

Participants completed questionnaires measuring initial trust in automation (TIA), interpersonal trust (ITS), and personality traits (EPQ). Descriptive statistics are summarized in Table 4. Mean scores for the sample were as follows: TIA (*M* = 50.67, *SD* = 12.45), ITS (*M* = 68.66, *SD* = 7.55), EPQ-P (*M* = 4.57, *SD* = 2.96), EPQ-E (*M* = 13.83, *SD* = 3.75), EPQ-N (*M* = 2.87, *SD* = 2.68), and EPQ-L (*M* = 11.17, *SD* = 3.30).

To integrate the human–automation trust framework, we examined relationships between these stable traits and the key task-derived indices (behavioral, signal detection, and eye tracking). Pearson and Spearman correlation matrices are provided in Table 4. Consistent with theoretical expectations, TIA was negatively associated with ITS (Pearson *r* = −0.42, *p* = 0.021; Spearman *ρ* = −0.46, *p* = 0.011), and EPQ-N was positively associated with ITS (Pearson *r* = 0.39, *p* = 0.032).

Critically, however, TIA did not exhibit reliable associations with the situational calibration indices derived from performances and monitoring (e.g., ΔRT, Δ*d′*, ΔAOI fixation duration; all computed as semi–auto). The correlations were small to moderate and non-significant (all |*r*| ≤ 0.34, *ps* ≥ 0.067). Furthermore, the three focal calibration indices—ΔAOI monitoring, Δ*d′*, and ΔRT—were largely independent of each other (|*r*| ≤ 0.14, all *ps* ≥ 0.45). This suggests that the calibration expressed at the monitoring, sensitivity, and speed levels represents complementary facets of situational behavior rather than a single unitary “situational trust” score.

#### 3.4.2. Mediation Model of Traits: EPQ-N → ITS → TIA

Given the robust associations between neuroticism (EPQ-N), interpersonal trust (ITS), and initial trust in automation (TIA), we tested a mediation model using PROCESS (Model 4; Hayes, 2017; 5000 bootstrap samples) to isolate the trait-level pathway. EPQ-N was entered as the predictor (*X*), ITS as the mediator (*M*), and TIA as the outcome (*Y*).

Results indicated that EPQ-N significantly predicted ITS: *B* = 0.19, *SE* = 0.08, *t*(28) = 2.26, *p* = 0.032, 95% CI [0.02, 0.37]. In turn, ITS significantly predicted TIA: *B* = −0.70, *SE* = 0.30, *t*(27) = −2.30, *p* = 0.029, 95% CI [−1.33, −0.08]. The indirect effect was significant (*ab* = −0.13, bootstrapped 95% CI [−0.399, −0.003]), whereas the total effect of EPQ-N on TIA was not significant (*c* = −0.10, *p* = 0.506), nor was the direct effect (*c′* = 0.04, *p* = 0.811). This pattern is consistent with an indirect-only mediation, indicating that neuroticism-related variance in initial trust was expressed through interpersonal trust rather than a direct pathway.

#### 3.4.3. Exploratory Cross-Index Associations

Because the situational indices did not form a single correlated chain, we conducted exploratory regression analyses to probe specific functional linkages between performance layers within the semi-automation block.

First, we examined the sensitivity–latency linkage. Semi-condition sensitivity (*d′* in semi; *z*-standardized) significantly predicted the semi–auto RT difference (ΔRT): *R*^2^ = 0.19, *F* (1, 28) = 6.72, *p* = 0.015. Higher discrimination in the semi condition was associated with a more negative ΔRT (i.e., a larger RT advantage for semi-automation relative to automation): *B* = −0.26, *SE* = 0.10, *t* (28) = −2.59, *p* = 0.015, 95% CI [−0.47, −0.06].

Second, we examined the bias–sensitivity linkage. The semi-condition response criterion (*β* in semi; *z*-standardized) significantly predicted the sensitivity increase (Δ*d′*): *R*^2^ = 0.16, *F* (1, 28) = 5.49, *p* = 0.026. A stricter response criterion was associated with a smaller sensitivity benefit: *B* = −0.39, *SE* = 0.17, *t* (28) = −2.34, *p* = 0.026, 95% CI [−0.73, −0.05].

Finally, we observed a trait–latency linkage. Interpersonal trust (ITS; *z*-standardized) significantly predicted the semi–manual RT difference: *R*^2^ = 0.15, *F* (1, 28) = 4.94, *p* = 0.035. Higher interpersonal trust was associated with a larger RT cost of semi-automation relative to manual control: *B* = 0.60, *SE* = 0.27, *t* (28) = 2.22, *p* = 0.035, 95% CI [0.05, 1.15].

Collectively, these analyses support a multi-level framing of trust: stable traits relate to initial trust via a definable pathway (EPQ-N → ITS → TIA), whereas situational trust expressions are multi-faceted (monitoring, sensitivity, and latency) and largely independent of questionnaire-based initial trust.

## 4. Discussion

The results of this study will be discussed in the context of the previous literature on trust in automation, focusing on how stable traits, initial trust, and situational trust interact over time. We will explore how stable individual differences, particularly personality traits and interpersonal trust, mediate trust calibration processes and suggest practical implications for HMI design in autonomous vehicles.

### 4.1. An Overview of Key Findings: The Cognitive Cost of Semi-Automation

Consistent with a human–automation trust calibration perspective, behavioral and gaze patterns suggested that participants allocated more monitoring resources and achieved better hazard discrimination under semi-automation than under full automation. Specifically, semi-automation yielded higher trial-level sensitivity (*d’*) for detecting hazards, alongside a substantially greater fixation duration on the mechanical-instrument AOI (32.09% vs. 7.26% under full automation).

However, this increased sensitivity came at a demonstrable cost. The pupil diameter, a reliable physiological proxy for cognitive workload and arousal (Lohani et al., 2019), was significantly larger in both automated conditions compared to manual driving. Furthermore, reaction times (RTs) did not improve with automation—manual driving remained significantly faster than both automated conditions. These findings indicate that semi-automation induces a “high-effort” monitoring state: drivers achieve better discrimination not by relying on the system to reduce their workload but by actively engaging in resource-intensive supervision.

### 4.2. Situational Trust as Multi-Layer Calibration

Following contemporary views that trust in automation is dynamic and context-dependent (Chiou & Lee, 2023; Kraus et al., 2020), this study operationalized situational calibration through multiple observable facets rather than a single proxy. The results support a multi-layer dissociation account: changes in monitoring allocation (ΔAOI), perceptual performance (Δ*d’*), and behavioral readiness (ΔRT) were largely independent of each other (*|r|* ≤ 0.14).

This indicates that “trust calibration” manifests distinctly across attentional, perceptual, and behavioral channels. Practically, an HMI that successfully increases visual monitoring (gaze) may not automatically translate into faster behavioral interventions (RT), suggesting that trust in information (believing the display) is distinct from reliance on action (taking control) (Azevedo-Sa et al., 2021).

### 4.3. The Trait-Level Pathway: The “Wary but Complacent” Paradox

At the stable trait level, we identified a meaningful pathway linking stable personality traits to both initial trust and situational behavior. First, a mediation analysis supported a pathway where neuroticism (EPQ-N) positively predicted interpersonal trust (ITS), which in turn negatively predicted initial trust in automation (TIA). This suggests that high-neuroticism individuals may project social wariness onto automated systems (Chien et al., 2016).

However, a critical paradox emerged when linking these traits to actual driving performance. Despite reporting lower initial trust, individuals with higher interpersonal trust (ITS) exhibited a significantly larger reaction time deficit (slower response) in the semi-automated condition relative to manual driving (*B* = 0.60, *p* = 0.035).

This finding illuminates a dissociation between subjective attitudes and objective reliance, a phenomenon increasingly documented in recent human autonomy teaming research (Petkac et al., 2025). While high-ITS individuals verbally expressed distrust (likely a mechanism of social anxiety), their behavioral physiology betrayed an implicit reliance on the “partner.” This aligns with the theory of “social loafing” in AI interaction: individuals who are prone to trusting social partners may subconsciously treat the automation as a teammate, leading to a diffusion of responsibility and reduced cognitive effort—even if they intellectually question the system’s capability (Elshan et al., 2025; Cymek et al., 2023). Essentially, these drivers fell into a “social complacency” trap: their stable tendency (ITS) to rely on others persisted despite their initial specific skepticism of the machine, manifesting as a dangerous form of behavioral lethargy.

### 4.4. Linking Perception and Decision Policy

Although a single sequential calibration chain was not supported, regression models clarified the performance trade-offs under semi-automation. Higher sensitivity (*d’*) was associated with reduced reaction time costs, implying that better hazard discriminators could mitigate the latency penalty of automation.

However, a stricter decision criterion (*β*)—representing a conservative response policy—predicted smaller sensitivity gains. These findings align with the “complacency-bias” framework, suggesting that when drivers adopt a conservative policy (hesitance to intervene), it can negate the perceptual benefits provided by the semi-automated interface (Merritt et al., 2019).

### 4.5. Theoretical and Practical Contributions

Theoretically, this work contributes to the literature by explicitly separating stable traits and initial trust from situational calibration indices. We demonstrate that these indices do not collapse onto a single latent dimension; rather, trust is expressed through parallel channels (gaze, physiology, and response time) that vary in their sensitivity to task constraints. Specifically, our findings advance the work of Molnar et al. (2018), who previously found no significant link between general trust propensities and handover response times. By identifying interpersonal trust (ITS) as a significant predictor of RT cost (*B* = 0.60), we provide evidence of a specific “social complacency” mechanism that previous models missed. Furthermore, we answer the specific call by De Cet et al. (2024) to incorporate physiological data; our pupillometry results demonstrate that “trusting” behavior in semi-automation is cognitively expensive, a nuance lost in binary intervention measures.

Practically, these findings are directly relevant to Level 2/3 HMI design. The observation that semi-automation improved discrimination but increased physiological loads and failed to improve RTs suggests that current monitoring prompts are insufficient. Future HMIs should work to achieve the following:

- Facilitate Transitions: Pair monitoring prompts with multimodal cues (auditory/haptic) to facilitate faster motor responses.
- Address Individual Differences: Since high interpersonal trust predicts slower reactions, adaptive systems could profile users during onboarding and provide more salient alerts for “high-trust” individuals to counteract the potential complacency identified in our trait–behavior analysis.

### 4.6. Limitations and Future Directions

The interpretation of our findings requires an acknowledgement of specific limitations.

- Interface Confound: As noted in recent HMI research, the automation level in our design was confounded with the interface presentation mode (HUD vs. Tablet). Therefore, the increased fixation on the instrument cluster may reflect both the monitoring requirement and the physical salience of the display. Future studies should orthogonally manipulate automation logic and the display location.
- Order Effects: The experimental conditions were administered in a fixed sequence (auto → manual → semi). Additionally, the intervening manual block may have served as a washout period, reducing direct carry-over effects between the two automated conditions. While statistical models controlled for the trial order, residual order effects (e.g., fatigue or practice) cannot be completely ruled out.
- Sample Characteristics: The sample was relatively small (N = 30) and consisted exclusively of male participants. Although this homogeneity limits generalizability, it reduced the variance related to age and gender, allowing for a focused examination of initial trust formation in novice users. Given known gender differences in risk perception, these results should be generalized with caution.
- Measurement: We relied on questionnaires for trait measurement; future work should integrate implicit association tests to capture implicit trust attitudes that bypass social desirability biases.

Finally, it is important to acknowledge a limitation regarding the causality of the findings. As the individual differences (personality and interpersonal trust) were measured traits rather than experimentally manipulated variables, the current data are correlational in nature. Therefore, while significant predictive associations between traits and trust behaviors were observed, causal links cannot be definitively established. Future research utilizing longitudinal designs or experimental interventions is needed to further explore the causal directionality of these relationships.

## 5. Conclusions

This study examined the architecture of human–automation trust across manual, fully automated, and semi-automated driving. We found that, within our sample of young male drivers, manual driving produced the fastest hazard responses, whereas semi-automation enhanced discrimination sensitivity but at the cost of increased monitoring demands and physiological workloads. Critically, the findings suggest that situational trust can be conceptualized as a multi-layer calibration process—expressed through dissociated attentional, perceptual, and behavioral mechanisms—rather than a single monolithic indicator.

Additionally, we identified a trait-level risk factor: high interpersonal trust is not only associated with lower initial trust (expectations) but also paradoxically predicts slower reaction times during semi-automated driving. This suggests a “wary but complacent” mechanism, likely driven by social loafing effects (Cymek et al., 2023). These insights advocate for multimodal assessment frameworks in the design and evaluation of future semi-automated driving systems.

## Figures and Tables

**Table 1:** Means and standard deviations of brake count and reaction time.

Automation Level	Brake Count	Reaction Time (s)
Fully automated	8.73 ± 5.12	3.95 ± 0.71
Semi-automated	7.93 ± 2.13	3.45 ± 0.68
Manual	20.00 ± 0.00	2.60 ± 0.26

Note. N = 30. Brake counts indicate the number of braking responses within each 20-trial block (range: 0–20). Reaction time (RT) is in seconds and was computed for brake trials (brake = 1). Values represent raw means without correction for trial order.

**Table 2:** Means and standard deviations of SDT indices in the automated conditions.

Automation Level	*d′*	*β*
Fully automated	0.28 ± 0.80	1.44 ± 1.21
Semi-automated	0.80 ± 0.66	0.96 ± 0.25

Note. N = 30. *d′* and *β* were computed at the participant level using the standard 1/(2N) correction for extreme hit/false-alarm rates (signal: hazard = 1, N = 5; noise: hazard = 0, N = 15). Inferential tests are based on the trial-level probit GLMM reported in Section 3.2.

**Table 3:** Means and standard deviations of eye-tracking measures across automation levels.

Automation Level	Overall Pupil Diameter (mm)	Mechanical-Instrument AOI Fixation Duration (%)
Fully automated	3.80 ± 0.50	7.26 ± 8.10
Semi-automated	3.68 ± 0.51	32.09 ± 17.39
Manual	2.84 ± 1.11	0.36 ± 0.66

Note. *N* = 30. Overall pupil diameter was calculated as the mean pupil diameter per condition. Mechanical-instrument AOI fixation duration (%) was computed as (AOI fixation duration/total fixation duration) × 100 within each condition.

**Table 4:** Correlations between trait measures and situational indices.

	1	2	3	4	5	6	7	8	9	10	11	12
1. TIA	—	−0.46 *	−0.09	0.04	−0.08	0.04	−0.02	0.07	0.05	−0.29	−0.14	0.01
2. ITS	−0.42 *	—	0.15	−0.04	0.39 *	−0.06	0.02	−0.12	0.09	0.19	0.34	−0.06
3. EPQ-P	−0.08	0.19	—	0.02	0.30	−0.54 **	−0.32	−0.36	0.37 *	0.20	0.45 *	−0.03
4. EPQ-E	0.04	−0.08	−0.03	—	−0.30	−0.03	−0.34	−0.30	−0.12	0.24	0.26	−0.17
5. EPQ-N	−0.13	0.39 *	0.38 *	−0.28	—	−0.35	0.11	−0.16	0.32	−0.16	0.08	−0.13
6. EPQ-L	−0.04	−0.11	−0.48 **	−0.12	−0.33	—	0.00	0.23	0.01	−0.26	−0.17	0.06
7. Δ*d′* (semi–auto)	−0.10	0.05	−0.30	−0.29	0.12	0.05	—	0.04	−0.11	−0.23	−0.38 *	−0.20
8. ΔAOI fixation duration (%) (semi–auto)	−0.03	−0.10	−0.25	−0.40 *	−0.19	0.16	−0.01	—	0.13	−0.29	−0.18	0.02
9. ΔRT (semi–auto)	0.01	0.07	0.39 *	−0.07	0.29	−0.02	−0.14	0.14	—	−0.45 *	0.40 *	−0.11
10. ΔRT (auto–manual)	−0.25	0.28	0.08	0.16	−0.09	−0.19	−0.13	−0.24	−0.55 **	—	0.52 **	−0.02
11. ΔRT (semi–manual)	−0.27	0.39 *	0.46 *	0.12	0.18	−0.23	−0.29	−0.14	0.33	0.60 **	—	−0.03
12. ΔPupil diameter (semi–auto)	−0.05	−0.03	−0.08	−0.17	−0.20	−0.03	−0.09	−0.02	−0.19	−0.03	−0.22	—

Note. N = 30. Pearson correlations are presented below the diagonal; Spearman’s rho is presented above the diagonal. * *p* < 0.05. ** *p* < 0.01 (two-tailed).

## Data Availability

The data utilized in this study is not publicly available due to confidentiality and privacy obligations. However, data access may be granted upon reasonable request to the corresponding author, subject to appropriate ethical approvals.
